# Association between covid-19 vaccination, SARS-CoV-2 infection, and risk of immune mediated neurological events: population based cohort and self-controlled case series analysis

**DOI:** 10.1136/bmj-2021-068373

**Published:** 2022-03-16

**Authors:** Xintong Li, Berta Raventós, Elena Roel, Andrea Pistillo, Eugenia Martinez-Hernandez, Antonella Delmestri, Carlen Reyes, Victoria Strauss, Daniel Prieto-Alhambra, Edward Burn, Talita Duarte-Salles

**Affiliations:** 1 Centre for Statistics in Medicine, Nuffield Department of Orthopaedics, Rheumatology, and Musculoskeletal Sciences (NDORMS), University of Oxford, Oxford OX3 7LD, UK; 2 Fundació Institut Universitari per a la recerca a l’Atenció Primària de Salut Jordi Gol i Gurina (IDIAPJGol), Barcelona, Spain; 3 Universitat Autònoma de Barcelona, Bellaterra (Cerdanyola del Vallès), Barcelona, Spain; 4 Neurology Department, Hospital Clinic de Barcelona and University of Barcelona, Barcelona, Spain; 5 Department of Medical Informatics, Erasmus University Medical Center, Rotterdam, Netherlands

## Abstract

**Objective:**

To study the association between covid-19 vaccines, SARS-CoV-2 infection, and risk of immune mediated neurological events.

**Design:**

Population based historical rate comparison study and self-controlled case series analysis.

**Setting:**

Primary care records from the United Kingdom, and primary care records from Spain linked to hospital data.

**Participants:**

8 330 497 people who received at least one dose of covid-19 vaccines ChAdOx1 nCoV-19, BNT162b2, mRNA-1273, or Ad.26.COV2.S between the rollout of the vaccination campaigns and end of data availability (UK: 9 May 2021; Spain: 30 June 2021). The study sample also comprised a cohort of 735 870 unvaccinated individuals with a first positive reverse transcription polymerase chain reaction test result for SARS-CoV-2 from 1 September 2020, and 14 330 080 participants from the general population.

**Main outcome measures:**

Outcomes were incidence of Bell’s palsy, encephalomyelitis, Guillain-Barré syndrome, and transverse myelitis. Incidence rates were estimated in the 21 days after the first vaccine dose, 90 days after a positive test result for SARS-CoV-2, and between 2017 and 2019 for background rates in the general population cohort. Indirectly standardised incidence ratios were estimated. Adjusted incidence rate ratios were estimated from the self-controlled case series.

**Results:**

The study included 4 376 535 people who received ChAdOx1 nCoV-19, 3 588 318 who received BNT162b2, 244 913 who received mRNA-1273, and 120 731 who received Ad26.CoV.2; 735 870 people with SARS-CoV-2 infection; and 14 330 080 people from the general population. Overall, post-vaccine rates were consistent with expected (background) rates for Bell’s palsy, encephalomyelitis, and Guillain-Barré syndrome. Self-controlled case series was conducted only for Bell’s palsy, given limited statistical power, but with no safety signal seen for those vaccinated. Rates were, however, higher than expected after SARS-CoV-2 infection. For example, in the data from the UK, the standardised incidence ratio for Bell’s palsy was 1.33 (1.02 to 1.74), for encephalomyelitis was 6.89 (3.82 to 12.44), and for Guillain-Barré syndrome was 3.53 (1.83 to 6.77). Transverse myelitis was rare (<5 events in all vaccinated cohorts) and could not be analysed.

**Conclusions:**

No safety signal was observed between covid-19 vaccines and the immune mediated neurological events of Bell’s palsy, encephalomyelitis, Guillain-Barré syndrome, and transverse myelitis. An increased risk of Bell’s palsy, encephalomyelitis, and Guillain-Barré syndrome was, however, observed for people with SARS-CoV-2 infection.

## Introduction

As of 13 January 2022, the covid-19 pandemic has resulted in more than 5.5 million deaths worldwide. After the rapid development of anti-SARS-CoV-2 vaccines, 9.2 billion doses have been administered through national vaccination programmes.^1^ To date, five vaccines against SARS-CoV-2 have received a conditional marketing authorisation by the European Medicines Agency. These include two mRNA vaccines: BNT162b2 (Pfizer-BioNTech) and mRNA-1273 (Moderna); two viral vector vaccines: ChAdOx1 nCoV-19 (Oxford-AstraZeneca) and Ad.26.COV2.S (Janssen/Johnson & Johnson); and one adjuvanted, recombinant spike protein nanoparticle vaccine: NVX-CoV2373 (Novavax).^2^ All of these vaccines have shown high efficacy in preventing severe covid-19 and acceptable safety profiles in clinical trials.^3^ ^4^ ^5^ ^6^ ^7^ However, potential adverse events related to these new vaccines have been reported, and continuous vaccine safety surveillance is needed as mass immunisation against covid-19 continues.

More recently, concerns have been raised about immune mediated neurological disorders post-covid-19 vaccination. Owing to reports of people developing Guillain-Barré syndrome after vaccination with Ad.26.COV2.S (108 of 21 million people vaccinated as of late June 2021),^8^ and ChAdOx1 nCoV-19 (833 people of 592 million doses administered as of late July 2021),^9^ ^10^ the EMA listed Guillain-Barré syndrome as a rare side effect related to these vaccines. Guillain-Barré syndrome has also been associated with mRNA vaccines in a few people.^11^ ^12^ In addition, Bell’s palsy, encephalomyelitis, and transverse myelitis events have been described in case series studies after covid-19 vaccination with both viral vector and mRNA vaccines.^13^ ^14^ ^15^ ^16^ ^17^ Although these events are not necessarily due to covid-19 vaccines, the temporal association between the events and vaccination warrants robust post-vaccination surveillance. Large scale epidemiological studies are required to determine whether covid-19 vaccination increases the risks of these events above background rates in the general population.

We leveraged large routinely collected datasets including millions of vaccinated people in the UK and Spain to study the potential association between covid-19 vaccines and the short term risk of developing Bell’s palsy, encephalomyelitis, Guillain-Barré syndrome, and transverse myelitis. To place these risks in context, we also studied the association between SARS-CoV-2 infection and risk of these immune mediated neurological events.

## Methods

### Data sources

For this study we used data from primary care records in both the UK and Spain. The Clinical Practice Research Datalink (CPRD) AURUM contains routinely collected data from primary care practices in the UK,^18^ ^19^ representing 20% of the current UK population.^20^ Data from Spain came from the Information System for Research in Primary Care (SIDIAP; www.sidiap.org), a primary care database that covers 80% of the population in Catalonia, Spain, and is linked at an individual level to hospital data. These hospital data included information from all public and private hospitals in Catalonia (Conjunt Mínim Bàsic de Dades d’Alta Hospitalària, CMBD-AH).^21^ Both databases have been mapped to the Observational Medical Outcomes Partnership (OMOP) common data model,^22^ which allowed the same analytical code to be run against both datasets without the need to share patient level data.

### Study participants

The populations of interest were individuals who had received at least one dose of a covid-19 vaccine and people with SARS-CoV-2 infection. Vaccination cohorts were constructed of people vaccinated according to the product (ChAdOx1 nCoV-19, BNT162b2, mRNA-1273, or Ad.26.COV2.S) and dose administered (first or second dose, with only a single dose cohort for Ad.26.COV2.S as this vaccine is comprises a one dose regimen). Ad26.COV2.S and mRNA-1273 cohorts were only available in SIDIAP. Vaccinated individuals were required to have received their first dose between the start of the vaccination campaign in each country (8 December 2020 in the UK, 27 December 2020 in Spain) and one week before the end of data availability of each database (9 May 2021 for CPRD AURUM, 30 June 2021 for SIDIAP), with the vaccination date used as index date. We excluded those who received more than one brand of a covid-19 vaccine. For the second dose cohorts, participants were required to have received their second dose in prespecified intervals after the first dose. For both databases, the interval allowed between doses of two dose regimens (except Ad26.COV2.S) was 14 to 180 days. The SARS-CoV-2 cohort included people with a first positive reverse transcription polymerase chain reaction (RT-PCR) test result or antigen test result between 1 September 2020 and one week before the end of data availability of each database, with the test date used as index date. Data from both RT-PCR and antigen tests were available in SIDIAP, whereas only RT-PCR test results were available in CPRD AURUM. From the SARS-CoV-2 cohort we excluded individuals vaccinated against covid-19 before infection. We also identified a background population cohort that included all individuals registered in CPRD AURUM and SIDIAP as of 1 January 2017 (index date).

All study participants were required to be 18 years or older and to have at least 365 days of data availability before their index date. For each specific outcome, we excluded those who had experienced the outcome in the year before the index date. For each cohort, we followed participants from the index date until the earliest of end of follow-up (21 days for vaccinated people, 90 days for those with a diagnosis of covid-19, and 31 December 2019 for the general population cohort), first occurrence of the adverse event, end of data availability, or until transference out of the database or death. For cohorts that had received a first vaccine dose, we also censored follow-up if a second dose was observed before 21 days.

### Events of interest

The events of interest were four immune mediated neurological disorders prespecified as potential adverse events of special interest for covid-19 vaccine safety: Bell’s palsy, encephalomyelitis, Guillain-Barré syndrome, and transverse myelitis.^2^ We identified these events using previously published clinical codes from electronic health records.^23^ Supplementary appendix table 1 provides details of the Systematized Nomenclature of Medicine (SNOMED) codes used to define the outcomes.

### Study design

**Fig 1:**
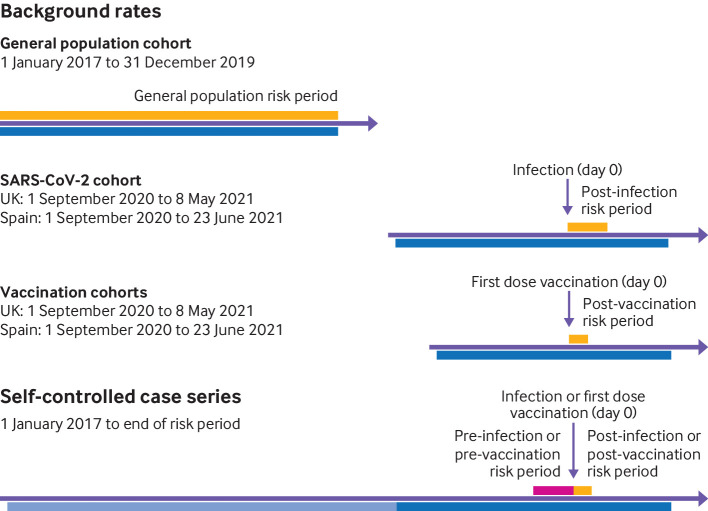
Study design. Potential risk period (dark blue) for vaccination cohorts was defined as time between the start of the vaccination campaign and one week before the end of data availability for each database (CPRD AURUM: 8 December 2020 to 2 May 2021; SIDIAP: 27 December 2020 to 23 June 2021). For the SARS-CoV-2 infected cohort, the potential risk period started on 1 September 2020. The baseline period for the self-controlled case series analysis (light blue) was defined from 1 January 2017 to 21 days before the day of vaccination or SARS-CoV-2 positive test result. The pre-risk period (pink) was defined as −21 to −1 days before vaccination or SARS-CoV-2 positive test result, and the risk period (orange) was defined as 1 to 21 days after vaccination and 1-90 days after a SARS-CoV-2 positive test result

Firstly, we used the historical rate comparison method (see fig 1). Incidence rates of each outcome in the vaccinated and SARS-CoV-2 cohorts were used as observed rates and compared with the expected background incidence rates estimated from the general population cohort. For the vaccinated cohorts, we estimated the rates during 1-21 days after a first vaccine dose (day 0). For the SARS-CoV-2 cohort we used a post-test period of 90 days.

Secondly, we used a self-controlled case series method. In this approach, only individuals who experience the outcome are included, with participants acting as their own controls and thereby eliminating time fixed confounding.^2^ Within person comparisons of event rates were made between the baseline period before vaccination and the period at risk of an outcome. We defined the at risk period as the 0-21 days after a first vaccine dose or after a SARS-CoV-2 positive test result. The 0-21 day period was subdivided into several prespecified time periods: 0, 1-7, 8-14, 15-21 days, as well as for the 1-21 day period overall. We considered events at day 0 separately, as these events might precipitate hospital admissions and subsequent covid-19 testing, which could result in a positive association between SARS-CoV-2 infection and the studied events.^25^ The risk period for the SARS-CoV-2 cohort was also extended to 90 days. The study period of the self-controlled case series analysis was defined from 1 January 2017 to 21 days after the first vaccine dose or 90 days after a positive test result.

### Statistical analysis

We characterised the participants in each cohort by personal characteristics, such as age and sex; comorbidities any time before vaccination; and recent drug use during the six months before the index date. Supplementary appendix table 2 shows the codes used for definitions of comorbidities and drug use. We estimated the observed rates during the 21 days after immunisation for the vaccinated cohorts and 90 days after testing for the SARS-CoV-2 cohort. Similarly, background rates were estimated for the general population from 1 January 2017 to 31 December 2019. We calculated crude incidence rates as the total number of events divided by the person time at risk per 100 000 person years. Indirect standardisation is used to account for differences between the age structure of the vaccinated or SARS-CoV-2 cohorts and the general population.^26^ Observed and expected rates were compared using standardised incidence ratios with corresponding 95% confidence intervals.

For the self-controlled case series analysis, only the first event was considered for each participant. Because events could potentially decrease the probability of being vaccinated, we removed a 21 day pre-risk period from the baseline period and reported separately. To assess potential for event dependent observation periods, we plotted a histogram of the time between the occurrence of the event and the end of observation for individuals censored and uncensored, and we calculated the number of deaths that occurred after each event (day 0 to day 7). Conditional Poisson regression models were fitted to estimate incidence rate ratios and 95% confidence intervals for each outcome,^27^ comparing the risk period with the baseline period. These models were estimated separately for each cohort of interest and were adjusted for age (in five year bands) and seasonality (four seasons). Self-controlled case series analyses were only conducted for comparisons with a ≤2 minimum detectable relative risk.^28^

We applied two sensitivity analyses to assess the impact of study design choices. Firstly, to exclude events potentially related to covid-19, in the vaccinated cohorts we excluded individuals infected with SARS-CoV-2 before the index date. For this, as RT-PCR tests were not routinely performed during the first wave of the pandemic, the covid-19 definition was broadened to include positive SARS-CoV-2 results (RT-PCR or antigen test) or a compatible covid-19 clinical code (see supplementary appendix table 3). Secondly, to include those with little previous use of healthcare, we also replicated the analyses after removing the one year before observation requirement for participants.

Any subgroups with fewer than five people were blinded and reported as less than five, following the requirements of information governance.

### Patient and public involvement

No patients or members of the public were directly involved in the design or analysis of the reported data. The independent scientific advisory committee responsible for the approval of our protocol involved patients in the evaluation of our data access application.

## Results

Figure 1 shows the study design and table 1 and table 2 show the distribution of personal characteristics, comorbidities, and recent drug use for each database and cohort. The study sample comprised 8 330 497 people who received at least one dose of a covid-19 vaccine (CPRD AURUM: 5 477 859; SIDIAP: 2 852 638), including 4 376 535 with ChAdOx1 nCoV-19, 3 588 318 with BNT162b2, 244 913 with mRNA-1273, and 120 731 with Ad26.COV2.S. Among those who received two vaccine doses (possible for all except Ad26.COV2.S), 3 745 017 completed the vaccination course (CPRD AURUM: 2 260 880; SIDIAP: 1 644 365), including 1 324 666 with ChAdOx1 nCoV-19, 2 420 351 with BNT162b2, and 160 228 with mRNA-1273. Among those vaccinated, 594 407 had covid-19 before the first dose. In addition, the study sample comprised 735 870 unvaccinated people with SARS-CoV-2 infection (CPRD AURUM: 447 233; SIDIAP: 288 637) and 14 330 080 people from the general population (CPRD AURUM: 9 651 568; SIDIAP: 4 678 512). Participants who received the first dose of any of the covid-19 vaccines were older (median age 56-64 years in CPRD AURUM, 51-62 years in SIDIAP) than the general population (median age 48 years in CPRD AURUM, 47 years in SIDIAP). In CPRD AURUM, participants with SARS-CoV-2 infection were younger than those vaccinated against covid-19 (median age 41 years). Vaccinated individuals also had more comorbidities than the general population, including autoimmune diseases, cancer, diabetes, obesity, heart disease, and renal impairment (table 1 and table 2).

**Table 1:** Baseline characteristics of study participants in UK’s Clinical Practice Research Datalink AURUM database

Characteristics	General population (n=9 651 568)	ChAdOx1 nCoV-19			BNT162b2		SARS-CoV-2 infection (n=447 233)
		First dose (n=3 782 401)	Second dose (n=1 093 812)		First dose (n=1 695 458)	Second dose (n=1 167 068)	
Median (IQR) age (years)	48 (33-63)	56 (47-66)	71 (59-76)		64 (49-76)	69 (52-78)	41 (30-54)
Men	4 825 624 (50.0)	1 829 719 (48.4)	482 007 (44.1)		711 217 (41.9)	462 061 (39.6)	204 798 (45.8)
Median (IQR) past observation time (years)	12.8 (4.9-23.7)	16.1 (6.7-27.6)	20.4 (8.2-32.4)		18.0 (7.1-30.1)	19.1 (7.5-31.3)	11.6 (4.5-22.2)
**Comorbidities**							
Autoimmune disease	198 170 (2.1)	105 128 (2.8)	47 582 (4.4)		62 384 (3.7)	45 003 (3.9)	8988 (2.0)
Malignant neoplastic disease	606 442 (6.3)	319 601 (8.4)	185 702 (17.0)		243 369 (14.4)	191 533 (16.4)	17 519 (3.9)
Diabetes mellitus	685 313 (7.1)	383 250 (10.1)	181 246 (16.6)		263 365 (15.5)	167 689 (14.4)	32 705 (7.3)
Obesity	349 425 (3.6)	190 790 (5.0)	67 163 (6.1)		98 771 (5.8)	61 431 (5.3)	20 436 (4.6)
Heart disease	827 039 (8.6)	413 054 (10.9)	232 267 (21.2)		319 053 (18.8)	236 329 (20.2)	28 603 (6.4)
Hypertensive disorder	1 761 421 (18.3)	915 008 (24.2)	449 684 (41.1)		592 676 (35.0)	445 346 (38.2)	59 638 (13.3)
Renal impairment	512 050 (5.3)	242 170 (6.4)	157 400 (14.4)		208 112 (12.3)	168 226 (14.4)	15 982 (3.6)
**Drug use (183 days before to four days before index date)**							
Systemic corticosteroids	545 205 (5.6)	187 454 (5.0)	77 668 (7.1)		103 444 (6.1)	73 838 (6.3)	18 932 (4.2)
Antithrombotics and anticoagulants	190 145 (2.0)	70 593 (1.9)	43 189 (3.9)		58 379 (3.4)	44 663 (3.8)	4388 (1.0)
Lipid modifying agents	290 710 (3.0)	137 925 (3.6)	71 958 (6.6)		95 123 (5.6)	69 719 (6.0)	9110 (2.0)
Antineoplastic and immunomodulating agents	170 815 (1.8)	40 343 (1.1)	14 601 (1.3)		25 199 (1.5)	18 078 (1.5)	9041 (2.0)

IQR=interquartile range.

**Table 2:** Baseline characteristics of study participants in Spain’s Information System for Research in Primary Care (SIDIAP) database

Characteristics	General population (n=4 678 512)	ChAdOx1 nCoV-19			BNT162b2			mRNA-1273		Ad26.COV2.S (n=120 731)	SARS-CoV-2 infection (n=288 637)
		First dose (n=594 134)	Second dose (n=230 854)		First dose (n=1 892 860)	Second dose (n=1 253 283)		First dose (n=244 913)	Second dose (n=160 228)		
Median (IQR) age (years)	47 (36-63)	62 (59-65)	60 (42-63)		56 (47-75)	72 (55-79)		53 (47-57)	54 (49-60)	51 (43-67)	46 (34-59)
Men	2 287 403 (48.9)	270 830 (45.6)	103 686 (44.9)		859 915 (45.4)	527 395 (42.1)		117 409 (47.9)	73 139 (45.6)	64 435 (53.4)	135 813 (47.1)
Median (IQR) past observation time (years)	11.0 (11.0-11.0)	15.3 (15.2-15.3)	15.5 (15.4-15.5)		15.3 (15.2-15.4)	15.3 (15.2-15.4)		15.4 (15.3-15.4)	15.4 (15.3-15.4)	15.4 (15.3-15.5)	14.9 (14.8-15.1)
**Comorbidities**											
Autoimmune disease	79 044 (1.7)	14 501 (2.4)	5075 (2.2)		49 029 (2.6)	40 899 (3.3)		13 012 (5.3)	10 776 (6.7)	2242 (1.9)	5857 (2.0)
Malignant neoplastic disease	339 004 (7.2)	60 074 (10.1)	18 504 (8.0)		241 140 (12.7)	220 040 (17.6)		38 401 (15.7)	32 964 (20.6)	9397 (7.8)	22 242 (7.7)
Diabetes mellitus	464 603 (9.9)	80 492 (13.5)	23 582 (10.2)		280 876 (14.8)	243 412 (19.4)		25 669 (10.5)	19 699 (12.3)	15 049 (12.5)	30 542 (10.6)
Obesity	863 167 (18.4)	161 047 (27.1)	50 787 (22.0)		502 689 (26.6)	396 021 (31.6)		55 920 (22.8)	38 727 (24.2)	30 154 (25.0)	66 933 (23.2)
Heart disease	568 547 (12.2)	87 455 (14.7)	26 640 (11.5)		403 280 (21.3)	367 372 (29.3)		30 494 (12.5)	24 061 (15.0)	15 827 (13.1)	37 800 (13.1)
Hypertensive disorder	1 139 337 (24.4)	200 957 (33.8)	56 782 (24.6)		701 276 (37.0)	628 916 (50.2)		60 731 (24.8)	46 745 (29.2)	33 102 (27.4)	65 030 (22.5)
Renal impairment	229 136 (4.9)	21 359 (3.6)	5650 (2.4)		194 837 (10.3)	187 662 (15.0)		14 622 (6.0)	12 998 (8.1)	4923 (4.1)	16 807 (5.8)
**Drug use (183 days before to four days before index date)**											
Systemic corticosteroids	258 775 (5.5)	32 229 (5.4)	11 213 (4.9)		123 581 (6.5)	98 008 (7.8)		20 231 (8.3)	15 854 (9.9)	7296 (6.0)	15 654 (5.4)
Antithrombotics and anticoagulants	110 809 (2.4)	16 968 (2.9)	4851 (2.1)		68 891 (3.6)	59 083 (4.7)		10 122 (4.1)	7838 (4.9)	3548 (2.9)	7618 (2.6)
Lipid modifying agents	80 561 (1.7)	18 339 (3.1)	5310 (2.3)		45 250 (2.4)	37 985 (3.0)		6292 (2.6)	4804 (3.0)	2934 (2.4)	4268 (1.5)
Antineoplastic and immunomodulating agents	59 360 (1.3)	7097 (1.2)	3277 (1.4)		24 460 (1.3)	18 331 (1.5)		7861 (3.2)	6455 (4.0)	1310 (1.1)	5046 (1.7)

IQR=interquartile range.

### Bell’s palsy

**Fig 2:**
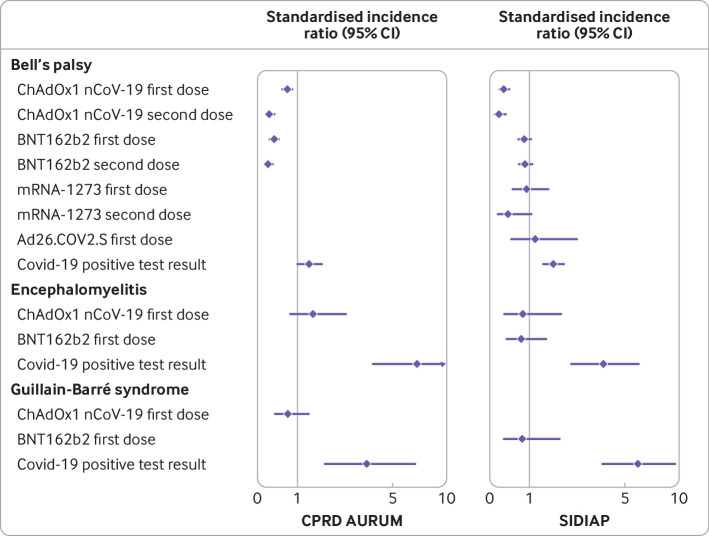
Standardised incidence ratios of immune mediated neurological disorders of special interest. CPRD AURUM=Clinical Practice Research Datalink AURUM (UK); SIDIAP=Information System for Research in Primary Care (Spain)

Within 21 days after a first dose of ChAdOx1 nCoV-19, Bell’s palsy was observed in 144 people: 117 observed in CPRD AURUM compared with 164.5 expected given background rates in the general population (standardised incidence ratio 0.71, 95% confidence interval 0.59 to 0.85; table 3). In SIDIAP, the corresponding values were 27 observed compared with 82.6 expected (0.33, 0.22 to 0.48; table 4). Thirty five patients (25 in CPRD AURUM; 10 in SIDIAP) were observed after the second dose of ChAdOx1 nCoV-19, resulting in a standardised incidence ratio of 0.26 (0.18 to 0.39) in CPRD AURUM and 0.21 (0.11 to 0.39) in SIDIAP (table 3, table 4, and fig 2). Bell’s palsy was observed in 46 and 24 patients after a first and second dose of BNT162b2 in CPRD AURUM, compared with 116.4 and 99.5 expected (0.40, 0.30 to 0.53, and 0.24, 0.16 to 0.36, respectively). In SIDIAP, rates of Bell’s palsy after BNT162b2 (first and second doses) and mRNA-1273 and Ad26.COV2.S first doses were similar to those expected, with equivalent standardised incidence ratios of 0.86 (0.70 to 1.04), 0.88 (0.71 to 1.08), 0.92 (0.54 to 1.55) and 1.15 (0.52 to 2.56). Incidence rate ratios could only be stratified by age and sex for Bell’s palsy, as it was the only event in which some of the strata had more than five occurrences. None of the strata showed significantly higher rates after vaccination in either CPRD AURUM or SIDIAP (see appendix figure 2). Rates of Bell’s palsy were higher than expected among those infected with SARS-CoV-2, with 53 and 93 events in the 90 days after an infection registered in CPRD AURUM and in SIDIAP, compared with 39.8 and 54.7 expected events, respectively. Equivalent standardised incidence ratios were 1.33 (1.02 to 1.74) in CPRD AURUM and 1.70 (1.39 to 2.08) in SIDIAP.

**Table 3:** Association between covid-19 vaccination or SARS-CoV-2 infection and occurrence of immune mediated neurological disorders of special interest in UK’s Clinical Practice Research Datalink AURUM database

Event by vaccine and dose	No of participants	Person years	Observed events	Expected events	Standardised incidence ratio (95% CI)
**Bell’s palsy**					
ChAdOx1 nCoV-19:					
First dose	3 776 803	384 250	117	164.5	0.71 (0.59 to 0.85)
Second dose	1 093 258	218 629	25	94.7	0.26 (0.18 to 0.39)
BNT162b2:					
First dose	1 693 453	275 333	46	116.4	0.40 (0.30 to 0.53)
Second dose	1 166 571	235 351	24	99.5	0.24 (0.16 to 0.36)
SARS-CoV-2 positive test result	446 851	106 342	53	39.8	1.33 (1.02 to 1.74)
**Encephalomyelitis**					
ChAdOx1 nCoV-19:					
First dose	3 778 358	384 420	11	7.6	1.45 (0.80 to 2.62)
BNT162b2:					
First dose	1 694 161	275 451	<5	5.4	
Second dose	1 167 028	235 447	<5	4.6	
SARS-CoV-2 positive test result	447 037	106 391	11	1.6	6.89 (3.82 to 12.44)
**Guillain-Barré syndrome**					
ChAdOx1 nCoV-19:					
First dose	3 778 430	384 427	11	14.9	0.74 (0.41 to 1.33)
Second dose	1 093 778	218 737	<5	9.5	
BNT162b2:					
First dose	1 694 167	275 452	<5	10.4	
Second dose	1 167 031	235 448	<5	9.0	
SARS-CoV-2 positive test result	447 029	106 390	9	2.6	3.53 (1.83 to 6.77)
**Transverse myelitis**					
ChAdOx1 nCoV-19:					
First dose	3 778 434	384 430	<5	7.6	
BNT162b2:					
First dose	1 694 190	275 456	<5	4.7	
SARS-CoV-2 positive test result	447 041	106 393	<5	1.9	

Events with fewer than five occurrences have been omitted for privacy reasons. No events were observed for omitted cohorts.

**Table 4:** Association between covid-19 vaccination or SARS-CoV-2 infection and occurrence of immune mediated neurological disorders of special interest in Spain’s Information System for Research in Primary Care (SIDIAP) database

Event by vaccine and dose	No of participants	Person years	Observed events	Expected events	Standardised incidence ratio (95% CI)
**Bell’s palsy**					
ChAdOx1 nCoV-19:					
First dose	592 365	89 055	27	82.6	0.33 (0.22 to 0.48)
Second dose	230 692	55 659	10	47.3	0.21 (0.11 to 0.39)
BNT162b2:					
First dose	1 890 434	102 623	100	116.7	0.86 (0.70 to 1.04)
Second dose	1 251 680	74 638	85	97.1	0.88 (0.71 to 1.08)
mRNA-1723:					
First dose	244 467	17 648	14	15.2	0.92 (0.54 to 1.55)
Second dose	159 995	12 563	5	11.3	0.44 (0.18 to 1.06)
Ad26.COV2.S:					
First dose	120 470	5,228	6	5.2	1.15 (0.52 to 2.56)
SARS-CoV-2 positive test result	288 030	66 603	93	54.7	1.70 (1.39 to 2.08)
**Encephalomyelitis**					
ChAdOx1 nCoV-19:					
First dose	592 843	89 121	5	6.1	0.82 (0.34 to 1.97)
Second dose	230 844	55 696	<5	3.4	
BNT162b2:					
First dose	1 892 409	102 738	9	11.7	0.77 (0.40 to 1.48)
Second dose	1 253 202	74 733	<5	10.3	
mRNA-1273:					
First dose	244 744	17 669	<5	1.2	
Ad26.COV2.S:					
First dose	120 588	5,233	<5	0.4	
SARS-CoV-2 positive test result	288 374	66 689	17	4.5	3.75 (2.33 to 6.02)
**Guillain-Barré syndrome**					
ChAdOx1 nCoV-19:					
First dose	592 860	89 123	<5	5	
BNT162b2:					
First dose	1 892 423	102 739	5	6.3	0.79 (0.33 to 1.91)
Second dose	1 253 201	74 733	<5	5.3	
mRNA-1273:					
Second dose	160 213	12,58	<5	0.7	
SARS-CoV-2 positive test	288 392	66 693	17	2.9	5.92 (3.68 to 9.53)
**Transverse myelitis**					
BNT162b2:					
First dose	1 892 510	102 744	<5	0.9	
mRNA-1723:					
Second dose	160 222	12 581	<5	0.1	

Events with fewer than five occurrences have been omitted for privacy reasons. No events were observed for omitted cohorts.

The self-controlled case series analysis was only sufficiently powered to study those with a first dose of BNT162b2, ChAdOx1 nCoV-19, and mRNA-1273 (in SIDIAP only), and those with SARS-CoV-2 infection (see supplementary appendix tables 4 and 5). There was some indication of event dependent observation periods (see supplementary appendix figure 1). In the self-controlled case series analysis, an adjusted incidence rate ratio for Bell’s palsy of 0.81 (0.66 to 0.98) was observed for ChAdOx1 nCoV-19 and 0.83 (0.61 to 1.10) for BNT162b2 between one and 21 days in CPRD AURUM (table 5). The values in SIDIAP were 0.92 (0.60 to 1.33) and 0.83 (0.66 to 1.02) between eight and 14 days (table 6). The adjusted incidence rate ratio for mRNA-1273 was 0.99 (0.54 to 1.64), and for SARS-CoV-2 infection was 1.82 (1.21 to 2.61). An increased risk of Bell’s palsy was observed on the day of a SARS-CoV-2 positive test result, and 1-21 days before and 1-7 days after the test result (table 5 and table 6).

**Table 5:** Incidence rate ratios (95% confidence intervals) for covid-19 vaccination or SARS-CoV-2 infection and occurrence of Bell’s palsy using self-controlled case series analysis in UK’s Clinical Practice Research Datalink AURUM database

	ChAdOx1 nCoV-19 first dose			BNT162b2 first dose			SARS-CoV-2 infection	
	No of events	Incidence rate ratio (95% CI)		No of events	Incidence rate ratio (95% CI)		No of events	Incidence rate ratio (95% CI)
Baseline	7403	Ref		4564	Ref		2335	Ref
Days pre-vaccination/pre-infection:								
−21 to −1	83	0.68 (0.54 to 0.84)		41	0.74 (0.54 to 1.00)		14	1.03 (0.58 to 1.68)
Day 0	5	0.83 (0.30 to 1.79)		<5			<5	
Days post-vaccination/post-infection:								
1-7	25	0.59 (0.38 to 0.85)		14	0.75 (0.42 to 1.22)		6	1.30 (0.52 to 2.65)
8-14	34	0.81 (0.56 to 1.11)		17	0.91 (0.54 to 1.41)		<5	
15-21	43	1.03 (0.75 to 1.37)		16	0.84 (0.49 to 1.33)		7	1.51 (0.64 to 2.93)
1-21	102	0.81 (0.66 to 0.98)		47	0.83 (0.61 to 1.10)		*	0.94 (0.51 to 1.55)
1-90							48	0.76 (0.56 to 1.01)

Events with fewer than five occurrences have been omitted for privacy reasons.

* Since event number <5 was blinded, the total number of events during the period was also blinded.

**Table 6:** Incidence rate ratios (95% confidence intervals) for covid-19 vaccination or SARS-CoV-2 infection and the occurrence of Bell’s palsy estimated using self-controlled case series analysis in in SIDIAP

	ChAdOx1 nCoV-19			BNT162b2			mRNA-1273			SARS-CoV-2 infection	
	No of events	Incidence rate ratio (95% CI)		No of events	Incidence rate ratio (95% CI)		No of events	Incidence rate ratio (95% CI)		No of events	Incidence rate ratio (95% CI)
Baseline	4588	Ref		9150	Ref		3791	Ref		3892	Ref
Days pre-vaccination/pre-infection:											
−21 to −1	12	0.44 (0.23 to 0.73)		74	0.69 (0.54 to 0.86)		11	0.80 (0.41 to 1.38)		44	2.93 (2.13 to 3.92)
Day 0	<5			<5			<5			8	11.13 (5.07 to 20.80)
Days post-vaccination/post-infection:											
1-7	7	0.77 (0.33 to 1.49)		22	0.62 (0.39 to 0.91)		<5			17	3.38 (2.01 to 5.28)
8-14	7	0.77 (0.33 to 1.50)		35	1.02 (0.72 to 1.40)		6	1.37 (0.54 to 2.79)		6	1.21 (0.48 to 2.47)
15-21	11	1.22 (0.63 to 2.10)		27	0.85 (0.57 to 1.22)		<5			<5	
1-21	25	0.92 (0.60 to 1.33)		84	0.83 (0.66 to 1.02)		*	0.99 (0.54 to 1.64)		*	1.82 (1.21 to 2.61)
1-90										77	1.31 (1.03 to 1.64)

Events with fewer than five occurrences have been omitted for privacy reasons.

* Since event number <5 was blinded, the total number of events during the period was also blinded.

### Encephalomyelitis

Overall, 16 encephalomyelitis events were observed after a first dose of ChAdOx1 nCoV-19: standardised incidence ratio 1.45 (0.80 to 2.62) in CPRD AURUM and 0.82 (0.34 to 1.97) in SIDIAP (table 3 and table 4). Nine events were observed after a first dose of BNT162b2 in SIDIAP (0.77, 0.40 to 1.48). No events were reported after a second dose of mRNA-1273, and fewer than five occurred among the remaining vaccination cohorts. Conversely, 28 encephalomyelitis events occurred after SARS-CoV-2 infection: 11 observed to 1.6 expected (6.89, 3.82 to 12.44) in CPRD AURUM and 17 observed to 4.5 expected (3.75, 2.33 to 6.02) in SIDIAP.

### Guillain-Barré syndrome

In CPRD AURUM, 11 Guillain-Barré syndrome events were observed after a first dose of ChAdOx1 nCoV-19 and fewer than five after a second dose. The corresponding values in SIDIAP were fewer than five and zero events. Five or fewer events were observed after the first and second dose of BNT162b2 and the second dose of mRNA-1723, with no events after the first dose of mRNA or Ad26.COV2.S. However, 26 events occurred after SARS-CoV-2 infection: nine observed to 2.6 expected (3.53, 1.83 to 6.77) events in CPRD AURUM and 17 observed to 2.9 expected (5.92, 3.68 to 9.53) events in SIDIAP.

### Transverse myelitis

Transverse myelitis events were only observed after the first dose of ChAdOx1 nCoV-19 (in CPRD AURUM), first dose of BNT162b2 (in CPRD AURUM and SIDIAP) and second dose of mRNA-1723 (in SIDIAP), with fewer than five events. Fewer than five events were also observed after SARS-CoV-2 infection in CPRD AURUM.

### Sensitivity analyses

We found similar results in sensitivity analysis after excluding individuals with previous covid-19 among those vaccinated, removing the requirement for at least one year of history in all cohorts (see supplementary appendix table 6). We obtained similar results after reducing the follow-up period from 90 to 21 days in the SARS-CoV-2-infected cohorts, although standardised incidence ratios were higher and confidence intervals wider than those obtained in the main analysis. The first sensitivity analysis was also performed in the self-controlled case series, with similar results obtained to those of the main analysis (see supplementary appendix table 7). The results from primary and secondary analyses are available at https://livedataoxford.shinyapps.io/VaxAEsNeuroimmune.

### Hospital admissions and deaths after outcome events

In SIDIAP, the percentage of outcome events associated with hospital admission ranged from 35.9% for Bell’s palsy to 86.3% for encephalomyelitis (see supplementary appendix table 8). Deaths in the first week after an event occurred in less than 0.5% participants across both databases (see supplementary appendix table 9). 

## Discussion

In this study, which included more than eight million vaccine recipients from two countries, we observed no safety signal between BNT162b2, ChAdOx1 nCoV-19, mRNA-1273, or Ad26.COV2.S and risk of developing the immune mediated neurological events of Bell’s palsy, encephalomyelitis, Guillain-Barré syndrome, and transverse myelitis. An increased risk of Bell’s palsy, encephalomyelitis, and Guillain-Barré syndrome was, however, observed in two cohorts of 447 233 and 288 637 people with SARS-CoV-2 infection.

### Findings in context

Immune mediated neurological disorders have been identified as adverse events of special interest by regulators, such as the Food and Drug Administration in the US and the EMA in Europe, and organisations such as the Brighton Collaboration. These adverse events of special interest have been closely monitored during immunisation campaigns,^23^ ^29^ ^30^ and several severe neurological disorders were reported as rare adverse events during the first clinical trials of covid-19 vaccines.^4^ ^5^ ^31^ ^32^ ^33^ ^34^ ^35^ Although a causal relationship was not established, the recommendation for further monitoring has led to the publication of many case reports worldwide.^36^

However, research findings from observational studies on the association between covid-19 vaccines and immune mediated neurological events have been mixed. For mRNA vaccines, for example, although two studies underpinned by safety surveillance databases found no association between mRNA vaccines and Bell’s palsy,^37^ ^38^ two others observed an increased risk,^39^ ^40^ with the case series and nested case-control study from Hong Kong also reporting an increased risk for recipients of an inactivated vaccine (CoronaVac; Sinovac Biotech). Another study from Hong Kong, however, found no such increased risk when comparing 1.1 million recipients of BNT162b2 or CoronaVac with 2.7 million unvaccinated individuals.^41^ Our findings are in line with this study, with no safety signal seen for either BNT162b2 or mRNA-1273.

Although a study of more than 20 million people vaccinated with ChAdOx1 nCoV-19 and 12 million vaccinated with BNT162b2 in the UK similarly did not observe any increased risk of hospital admissions for neurological events with BNT162b2, it did find an association for ChAdOx1 nCoV-19.^42^ For example, the incidence rate ratios in the self-controlled case series analysis for Bell’s palsy and Guillain-Barré syndrome were 1.3 (1.1 to 1.6) and 2.9 (2.2 to 3.9), respectively, 15-21 days after vaccination. Moreover, this association between ChAdOx1 nCoV-19 and Guillain-Barré syndrome was subsequently confirmed using analogous methods with Scottish data.^42^ In our study we found no such safety signals for ChAdOx1 nCoV-19. Meanwhile, in another study comparing events reported in a vaccine surveillance system with background rates, Ad26.COV2.S was associated with increased risks of Guillain-Barré syndrome (observed to expected rate ratio of 4.2 (3.5 to 5.0) in the 42 days after vaccination).^43^ In our study, we observed no safety signal for Ad26.COV2.S, but this vaccine was only available for the data from Spain, and was the smallest vaccine cohort.

In line with our findings, several other studies have also reported increased risks of immune mediated neurological events after SARS-CoV-2 infection.^42^ ^44^ ^45^ One study, for example, found increased risks of Bell’s palsy; encephalitis, meningitis, and myelitis; and Guillain-Barré syndrome after SARS-CoV-2 infection.^42^ The increase in risks after SARS-CoV-2 infection much exceed any of the previously mentioned associations reported after vaccination.

### Limitations of this study

Our study has limitations. Firstly, CPRD AURUM only included primary care data from the UK. Therefore, diagnoses from inpatient settings might not be captured and the absolute risk could be underestimated. A previous study has, however, shown that CPRD AURUM captures immune mediated neurological disorders such as Guillain-Barré syndrome relatively accurately, even in the absence of linked hospital data.^46^ Meanwhile, the SIDIAP database did have patient level hospital linkage, and results were consistent between databases. Secondly, confounding by indication might affect the historical comparator method. Although we account for differences in age, individuals vaccinated also had more comorbidities than the comparator cohort and may well differ in other, unobserved characteristics. Thirdly, comparisons between the vaccinated and historical cohorts might be limited because of changes over time, including seasonal variations^47^ in outcomes and changes in exposure to other viral infections during the pandemic, which might have reduced the incidence of immune mediated neurological events.^48^ In addition, given public concerns over vaccine safety and complications from SARS-CoV-2 infection, individuals vaccinated against or infected with SARS-CoV-2 might have been more prone to seek care when showing symptoms. Although seasonality was not included in the historical comparison method, in our self-controlled case series analysis we adjusted for age and seasonality to control for time varying confounding. Fourthly, because we excluded individuals with a recent history of the same immune mediated neurological events, our results are not generalisable to people with chronic relapsing conditions such as multiple sclerosis or Devic’s disease. This exclusion criteria would also have led to a depletion-of-susceptible bias in the cohorts that received a second vaccine dose (because those who had an event after the first dose were excluded from the second dose cohort). Fifthly, we assessed outcomes using routinely recorded diagnoses without a formal adjudication, and outcome misclassification might therefore have been possible. However, we used code lists and algorithms for the identification of immune mediated neurological disorders previously published as part of a study on the background rates of covid-19 related adverse events of special interest.^23^

Our study focused on short term adverse events after vaccination, using similar time periods to other studies.^41^ ^42^ The time at risk after vaccination was defined according to the shortest recommended interval between first and second vaccine doses. Although the second dose is not a consideration for Ad26.COV2.S, we used the same time at risk to ensure comparability between the results. Therefore, further evidence is required to understand the long term adverse events of vaccination and SARS-CoV-2 infection. Larger cohorts are also needed to study the effect of vaccination on different age groups, particularly among younger populations. Moreover, although it is reassuring that so few outcomes of transverse myelitis were seen in this study, additional data might allow a more detailed study of this event.

### Conclusion

We found no safety signal for any of the studied immune mediated neurological events after vaccination against covid-19. Infection with SARS-CoV-2 was, however, associated with an increased risk of Bell’s palsy, encephalomyelitis, and Guillain-Barré syndrome.

**What is already known on this topic**

Spontaneous reporting indicated a potential association between immune mediated neurological disorders after covid-19 vaccination
The European Medicines Agency has listed Guillain-Barré syndrome as a rare side effect of exposure to viral vector vaccines
To date, however, the results from research into the risk of immune mediated neurological disorders after covid-19 vaccination have been mixed

**What this study adds**

Using routinely collected data from the UK and Spain, this study found no safety signal after covid-19 vaccination for the immune mediated neurological events of Bell’s palsy, encephalomyelitis, Guillain-Barré syndrome, and transverse myelitis
An increased risk of Bell’s palsy, encephalomyelitis, and Guillain-Barré syndrome was, however, seen after SARS-CoV-2 infection

## Data Availability

Patient level data cannot be shared without approval from data custodians owing to local information governance and data protection regulations. Analytical code and detailed definitions of algorithms for identifying the events are available in a GitHub repository (https://github.com/SIDIAP/VaxAEsNeuroimmune).

## Web extra

Extra material supplied by authors

## Notes

The lead authors (XL and BR) affirm that this manuscript is an honest, accurate, and transparent account of the study being reported; that no important aspects of the study have been omitted; and that any discrepancies from the study as planned (and, if relevant, registered) have been explained.

Dissemination to participants and related patient and public communities: We will disseminate a lay summary of our findings through our Twitter and other social media accounts.

Provenance and peer review: Not commissioned; externally peer reviewed.
