# Supplementary material for: Association between covid-19 vaccination, SARS-CoV-2 infection, and risk of immune mediated neurological events: population based cohort and self-controlled case series analysis
Source: BMJ. 2022 Mar 16;376:e068373. doi: 10.1136/bmj-2021-068373 (PMC8924704; doi:10.1136/bmj-2021-068373)
Supplement: Supplementary file 1 — Supplementary information: additional tables 1-9 and figures 1 and 2 [file lixi068373.ww.pdf]

SUPPLEMENTARY MATERIALS

Table 1. Codes used for the definition of study events.

Id	Code	Name	Vocabulary
**Bell's palsy**			
44784628	87651000119100	Facial palsy House-Brackmann grade VI	SNOMED
44782796	68151000119106	Facial palsy House-Brackmann grade V	SNOMED
44782795	68141000119109	Facial palsy House-Brackmann grade IV	SNOMED
44782794	68131000119100	Facial palsy House-Brackmann grade III	SNOMED
44782526	68121000119103	Facial palsy House-Brackmann grade II	SNOMED
42710044	449916008	Peripheral facial palsy	SNOMED
37396434	716243005	Sellars Beighton syndrome	SNOMED
37204553	783257005	Familial recurrent peripheral facial palsy	SNOMED
4091559	280816001	Facial palsy	SNOMED
4063208	164110008	O/E - cranial 7 -paralysis-UMN	SNOMED
4044386	230546006	Familial facial nerve palsy	SNOMED
4002030	11045000	Supranuclear facial nerve paralysis	SNOMED
3182576	12870001000004100	Partial seventh nerve palsy	Nebraska Lexicon
760893	12239661000119100	Bell's palsy of right side of face	SNOMED
760892	12239621000119100	Bell's palsy of left side of face	SNOMED
374923	193093009	Bell's palsy	SNOMED
**Transverse myelitis**			
4047628	230197009	Acute viral transverse myelitis	SNOMED
4044077	230378004	Acute non-infective transverse myelitis	SNOMED
4042204	230198004	Varicella transverse myelitis	SNOMED
443904	16631009	Transverse myelopathy syndrome	SNOMED
139803	47000000	Acute transverse myelitis	SNOMED
134330	425756000	Idiopathic transverse myelitis	SNOMED
**Guillain-Barré syndrome**			
37396786	716723000	Acute inflammatory demyelinating polyradiculoneuropathy	SNOMED
37396785	716722005	Acute motor sensory axonal neuropathy	SNOMED
37396086	715770009	Acute motor axonal neuropathy	SNOMED

37204541	783244002	Acute pure sensory neuropathy	SNOMED
37204369	783010003	Pharyngeal-cervical-brachial variant of Guillain-Barré syndrome	SNOMED
35624130	766722008	Paraparetic variant of Guillain-Barré syndrome	SNOMED
35623634	766049000	Acute sensory ataxic neuropathy	SNOMED
4164770	40956001	Guillain-Barré syndrome	SNOMED
4070552	1767005	Fisher's syndrome	SNOMED
374925	129131007	Acute infective polyneuritis	SNOMED
**Encephalomyelitis**			
46285143	963741000000101	Bacterial meningoencephalomyelitis	SNOMED
43530701	182961000119101	Acute disseminated encephalomyelitis following infectious disease	SNOMED
40480147	443153007	Toxic encephalomyelitis	SNOMED
36715541	721249007	Infection causing encephalomyelitis	SNOMED
36676712	773688007	Chronic lymphocytic inflammation with pontine perivascular enhancement responsive to steroids	SNOMED
35607947	763310000	Acute necrotizing encephalopathy of childhood	SNOMED
4330496	431520004	Inflammation of spinal cord due to toxin	SNOMED
4320933	70576008	Enteroviral encephalomyelitis	SNOMED
4310021	424761009	Paraneoplastic encephalomyelitis	SNOMED
4249574	72986009	Acute hemorrhagic leukoencephalitis	SNOMED
4242575	58766009	Granulomatous meningoencephalomyelitis	SNOMED
4233538	359686005	Van Bogaert's sclerosing leukoencephalitis	SNOMED
4228612	422089004	Encephalomyelitis associated with AIDS	SNOMED
4220014	397961002	Encephalomyelitis caused by lymphocytic choriomeningitis virus	SNOMED
4206037	55234004	Pyogranulomatous meningoencephalomyelitis	SNOMED
4190307	39367000	Inflammatory disease of the central nervous system	SNOMED
4176250	50776006	Allergic encephalomyelitis	SNOMED
4147498	267576008	Encephalitis, myelitis and encephalomyelitis	SNOMED
4120303	302811004	Progressive congenital rubella encephalomyelitis	SNOMED
4105201	192724008	Post mixed vaccination encephalitis	SNOMED
4105200	192711008	Post diphtheria vaccination encephalitis	SNOMED

4104690	192723002	Post hepatitis B vaccination encephalitis	SNOMED
4104546	29197009	Meningoencephalomyelitis	SNOMED
4103116	192721000	Post influenza vaccination encephalitis	SNOMED
4103115	192719005	Post mumps vaccination encephalitis	SNOMED
4103114	192718002	Post polio vaccination encephalitis	SNOMED
4103113	192716003	Post yellow fever vaccination encephalitis	SNOMED
4103112	192714000	Post rabies vaccination encephalitis	SNOMED
4103111	192712001	Post pertussis vaccination encephalitis	SNOMED
4103110	192709004	Post plague vaccination encephalitis	SNOMED
4103109	192708007	Post cholera vaccination encephalitis	SNOMED
4103108	192707002	Post paratyphoid vaccination encephalitis	SNOMED
4103107	192706006	Post typhoid vaccination encephalitis	SNOMED
4102199	192717007	Post measles vaccination encephalitis	SNOMED
4102198	192705005	Post BCG vaccination encephalitis	SNOMED
4100112	192722007	Post hepatitis A vaccination encephalitis	SNOMED
4100111	192720004	Post rubella vaccination encephalitis	SNOMED
4100110	192715004	Post typhus vaccination encephalitis	SNOMED
4100109	192713006	Post smallpox vaccination encephalitis	SNOMED
4100108	192710009	Post tetanus vaccination encephalitis	SNOMED
4098889	27040004	Experimental allergic encephalomyelitis	SNOMED
4042202	230188005	Post-influenza encephalitis	SNOMED
4035637	15316002	Simian B encephalomyelitis	SNOMED
3655306	860842004	Encephalomyelitis caused by Coxiella burnetii	SNOMED
3655305	860841006	Encephalomyelitis caused by bacterium	SNOMED
3655275	860805006	Encephalomyelitis caused by Neisseria meningitidis	SNOMED
3654623	838315004	Encephalomyelitis caused by Burkholderia	SNOMED
765827	432451000124103	Multiphasic acute disseminated encephalomyelitis	SNOMED
761989	2701000124105	Recurrent acute disseminated encephalomyelitis	SNOMED
443802	31367003	Postvaccinal encephalomyelitis	SNOMED
380322	186509002	Post Varicella encephalitis	SNOMED
379798	230199007	Post-infectious encephalomyelitis	SNOMED

379792	192704009	Post-immunization encephalitis	SNOMED
378143	45170000	Encephalitis	SNOMED
377487	111872008	Post measles encephalitis	SNOMED
375185	128191000	Rubella encephalomyelitis	SNOMED
374021	83942000	Acute disseminated encephalomyelitis	SNOMED
373189	62950007	Encephalomyelitis	SNOMED
372615	192727001	Post-infectious encephalitis	SNOMED
138965	41370002	Myelitis	SNOMED

SNOMED: Systematized Nomenclature of Medicine

Table 2. Codes used for the definition of comorbidities and medication use.

Id	Code	Name	Vocabulary
434621	85828009	Autoimmune disease	SNOMED
443392	363346000	Malignant neoplastic disease	SNOMED
201820	73211009	Diabetes mellitus	SNOMED
433736	414916001	Obesity	SNOMED
321588	56265001	Heart disease	SNOMED
316866	38341003	Hypertensive disorder	SNOMED
4030518	236423003	Renal impairment	SNOMED
21602722	H02	Corticosteroids for systemic use	ATC
21600961	B01A	Antithrombotic agents	ATC
21601853	C10	Lipid modifying agents	ATC
21601386	L	Antineoplastic and immunomodulating agents	ATC

SNOMED: Systematized Nomenclature of Medicine; ATC: Anatomical Therapeutic Chemical

Table 3. Medical codes used for the definition of compatible COVID-19 clinical code

Id	Code	Name	Vocabulary
45763724	700217006	Suspected coronavirus infection	SNOMED
44810277	878151000000108	Suspected coronavirus infection	SNOMED
44797466	431711000000107	[X]Coronavirus infection, unspecified	SNOMED
40479642	441590008	Pneumonia due to Severe acute respiratory syndrome coronavirus	SNOMED
40380828	187587009	Coronavirus as the cause of diseases classified to other chapters	SNOMED
37396171	715882005	Severe acute respiratory syndrome of upper respiratory tract	SNOMED
37311061	840539006	Disease caused by 2019-nCoV	SNOMED
37311060	840544004	Suspected disease caused by 2019-nCoV	SNOMED
37310287	1240531000000103	Myocarditis caused by 2019 novel coronavirus	SNOMED
37310286	1240541000000107	Infection of upper respiratory tract caused by 2019 novel coronavirus	SNOMED
37310284	1240561000000108	Encephalopathy caused by 2019 novel coronavirus	SNOMED
37310283	1240571000000101	Gastroenteritis caused by 2019 novel coronavirus	SNOMED
37310269	1240751000000100	Disease caused by 2019 novel coronavirus	SNOMED
37310268	1240761000000102	Suspected disease caused by 2019 novel coronavirus	SNOMED
37310254	1240521000000100	Otitis media caused by 2019 novel coronavirus	SNOMED
37016927	713084008	Pneumonia caused by Human coronavirus	SNOMED
4100065	27619001	Disease due to Coronaviridae	SNOMED
4092694	186758000	Coronavirus as the cause of diseases classified to other chapters	SNOMED
703441	1300721000000109	COVID-19 confirmed by laboratory test	SNOMED
703440	1300731000000106	Probable COVID-19 confirmed using clinical diagnostic criteria	SNOMED
439676	186747009	Coronavirus infection	SNOMED
320651	398447004	Severe acute respiratory syndrome	SNOMED

SNOMED: Systematized Nomenclature of Medicine

Table 4. Power calculation for the the self-controlled case series (SCCS)

	**CPRD AURUM**				**SIDIAP**			
	**Proportion time exposed**	**Total time**	**Events**	**MDRR**	**Proportion time exposed**	**Total time**	**Events**	**MDRR**
**ChAdOx1 first-dose**								
Bell's palsy	0.015	44230222	7403	1.29	0.0135	9086299	1925	1.62
Encephalomyelitis	0.0143	43035252	415	2.51	0.0145	8068323	111	4.31
Guillain Barre syndrome	0.0167	40030856	477	2.27	0.0136	9005332	101	4.67
Transverse myelitis	0.0142	59366705	304	2.85	0.0135	9045039	9	24.69
**BNT162b2 first-dose**								
Bell's palsy	0.012	30195714	4564	1.42	0.0134	31723854	7386	1.30
Encephalomyelitis	0.0121	23621329	260	3.25	0.0145	27732788	489	2.31
Guillain Barre syndrome	0.0125	28983007	313	2.96	0.0134	31797412	305	2.82
Transverse myelitis	0.0114	33884636	176	4.04	0.0128	33399967	50	7.22
**mRNA-1723 first-dose**								
Bell's palsy					0.0131	4684571	978	1.92
Encephalomyelitis					0.014	4275406	151	3.76
Guillain Barre syndrome					0.0132	4664538	71	5.75
Transverse myelitis					0.0133	4642457	51	6.98
**Ad26.COV2.S first-dose**								
Bell's palsy					0.0107	1922746	454	2.63
Encephalomyelitis					0.0116	1727694	22	13.34
Guillain Barre syndrome					0.0107	1910933	18	16.33

Transverse myelitis					0.0107	1917374	<5	
**SARS-CoV-2 infection (21 days at risk)**								
Bell's palsy	0.0046	10037690	2335	2.05	0.0135	4329031	1090	1.85
Encephalomyelitis	0.0049	9498944	127	7.90	0.0141	3954950	101	4.578
Guillain Barre syndrome	0.0046	9289326	147	7.43	0.0135	4328106	46	7.36
Transverse myelitis	0.0045	12357608	85	10.89	0.0134	4358694	6	35.99
**SARS-CoV-2 infection (90 days at risk)**								
Bell's palsy	0.0046	10037690	2335	2.05	0.0135	4329031	1090	1.85
Encephalomyelitis	0.0049	9498944	127	7.90	0.0141	3954950	101	4.58
Guillain Barre syndrome	0.0046	9289326	147	7.43	0.0135	4328106	46	7.36
Transverse myelitis	0.0045	12357608	85	10.89	0.0134	4358694	6	35.99

CPRD: Clinical Practice Research Datalink; SIDIAP: Information System for Research in Primary Care; MDRR: minimum detectable relative risks.

Table 5. Power calculation for the self-controlled case series (SCCS) sensitivity analyses

	CPRD AURUM				SIDIAP			
	**Proportion time exposed**	**Total time**	**Events**	**MDRR**	**Proportion time exposed**	**Total time**	**Events**	**MDRR**
**ChAdOx1 first-dose**								
Bell's palsy	0.0148	41838475	6935	1.30	0.0135	8383419	1761	1.65
Encephalomyelitis	0.0142	40517414	379	2.61	0.0145	7443524	106	4.41
Guillain Barre syndrome	0.0164	37867929	447	2.33	0.0136	8308627	89	4.50
Transverse myelitis	0.014	56213708	290	2.92	0.0135	8345559	9	24.68
**BNT162b2 first-dose**								
Bell's palsy	0.0118	29035280	4375	1.43	0.0134	28290593	6542	1.32
Encephalomyelitis	0.0119	22495388	244	3.37	0.0145	24707838	405	2.46
Guillain Barre syndrome	0.0123	27919905	289	3.08	0.0134	28348798	266	2.98
Transverse myelitis	0.0112	32515243	168	4.17	0.0128	29783708	45	7.71
**mRNA-1723 first-dose**								
Bell's palsy					0.0132	4162063	866	1.99
Encephalomyelitis					0.014	3794490	125	4.12
Guillain Barre syndrome					0.0132	4144053	62	6.22
Transverse myelitis					0.0133	4127669	41	8.01
**Ad26.COV2.S first-dose**								

Bell’s palsy					0.0107	1696564	396	2.77
Encephalomyelitis					0.0116	1523541	19	14.86
Guillain Barre syndrome					0.0108	1686084	13	21.10
Transverse myelitis					0.0107	1692484	<5	

CPRD: Clinical Practice Research Datalink; SIDIAP: Information System for Research in Primary Care; MDRR: minimum detectable relative risks.

Table 6A. Sensitivity analyses for the historical comparison analyses in the Clinical Practice Research Datalink (CPRD AURUM)

	N	Person-years	Observed events	Expected events	SIR (95% CI)
**Results reducing time at risk to 21 days among persons in the SARS-CoV-2-infected cohort**					
Bell's Palsy	446,851	25,54	15	9.6	1.57 (0.95 to 2.60)
Encephalomyelitis	447,037	25,551	<5	0.4	
Guillain Barré Syndrome	447,029	25,551	<5	0.6	
**Results excluding vaccinated persons with a history of COVID-19**					
Bell's Palsy					
ChAdOx1 first-dose	3,522,487	361,239	108	155.0	0.70 (0.58 to 0.84)
ChAdOx1 second-dose	1,029,876	206,264	24	89.5	0.27 (0.18 to 0.40)
BNT162b2 first-dose	1,599,332	261,317	43	110.7	0.39 (0.29 to 0.52)
BNT162b2 second-dose	1,099,149	221,961	22	94.0	0.23 (0.15 to 0.36)
Encephalomyelitis					
ChAdOx1 first-dose	3,523,932	361,399	9	7.2	1.25 (0.65 to 2.41)
BNT162b2 first-dose	1,599,992	261,429	<5	2.0	
BNT162b2 second-dose	1,099,577	222,051	<5	1.0	
Guillain Barré Syndrome					
ChAdOx1 first-dose	3,523,992	361,405	11	14.1	0.78 (0.43 to 1.41)
ChAdOx1 second-dose	1,030,356	206,365	<5	9.0	
BNT162b2 first-dose	1,600,002	261,43	<5	9.9	

BNT162b2 second-dose	1,099,583	222,052	<5	8.5	
Transverse Myelitis					
ChAdOx1 first-dose	3,523,987	361,407	<5	7.2	
BNT162b2 first-dose	1,600,021	261,434	<5	4.5	
**Results without requiring year of prior history**					
Bell's Palsy					
ChAdOx1 first-dose	4,045,751	409,047	124	174.2	0.71 (0.60 to 0.85)
ChAdOx1 second-dose	1,148,737	229,423	27	99.0	0.27 (0.19 to 0.40)
BNT162b2 first-dose	1,805,347	292,343	49	122.8	0.40 (0.30 to 0.53)
BNT162b2 second-dose	1,226,529	247,211	24	103.9	0.23 (0.15 to 0.34)
SARS-CoV-2 positive test	491,257	116,681	57	43.0	1.33 (1.02 to 1.72)
Encephalomyelitis					
ChAdOx1 first-dose	4,047,348	409,22	13	8.1	1.60 (0.93 to 2.76)
BNT162b2 first-dose	1,806,088	292,465	<5		
BNT162b2 second-dose	1,226,997	247,31	<5		
SARS-CoV-2 positive test	491,453	116,733	11	1.7	6.35 (3.52 to 11.47)
Guillain Barré Syndrome					
ChAdOx1 first-dose	4,047,416	409,228	12	15.5	0.77 (0.44 to 1.36)
BNT162b2 first-dose	1,806,095	292,466	<5		
BNT162b2 second-dose	1,227,001	247,31	<5		
SARS-CoV-2 positive test	491,447	116,732	11	2.7	4.01 (2.22 to 7.24)

Transverse Myelitis					
ChAdOx1 first-dose	4,047,425	409,231	<5		
BNT162b2 first-dose	1,806,120	292,47	<5		
SARS-CoV-2 positive test	491,459	116,735	<5		

Table 6B. Sensitivity analyses for the historical comparison analyses in the Information System for Research in Primary Care (SIDIAP)

	N	Person-years	Observed events	Expected events	SIR (95% CI)
**Results reducing time at risk to 21 days among persons in the SARS-CoV-2-infected cohort**					
Bell’s Palsy	288,03	16,39	36	13.5	2.66 (1.92 to 3.69)
Encephalomyelitis	288,374	16,411	10	1.1	8.84 (4.76 to 16.43)
Guillain Barré Syndrome	288,392	16,412	8	0.7	11.33 (5.66 to 22.65)
**Results excluding vaccinated persons with a history of COVID-19**					
Bell’s Palsy					
ChAdOx1 first-dose	556,581	85,327	23	79.2	0.29 (0.19 to 0.44)
ChAdOx1 second-dose	223,631	53,893	10	45.9	0.22 (0.12 to 0.41)
BNT162b2 first-dose	1,718,765	93,23	85	106.4	0.80 (0.65 to 0.99)
BNT162b2 second-dose	1,143,065	67,988	78	88.7	0.88 (0.70 to 1.10)
mRNA-1723 first-dose	219,459	15,971	12	13.9	0.87 (0.49 to 1.52)
mRNA-1723 second-dose	147,538	11,573	<5		
Ad26.COV2.S first-dose	108,031	4,692	5	4.7	1.06 (0.44 to 2.56)
Encephalomyelitis					
ChAdOx1 first-dose	557,017	85,389	5	5.8	0.86 (0.36 to 2.06)
ChAdOx1 second-dose	223,777	53,929	<5	3.3	
BNT162b2 first-dose	1,720,466	93,33	7	10.6	0.66 (0.31 to 1.38)
BNT162b2 second-dose	1,144,394	68,071	<5	9.4	

mRNA-1723 first-dose	219,706	15,99	<5	1.1	
Ad26.COV2.S first-dose	108,13	4,696	<5	0.4	
Guillain Barré Syndrome					
ChAdOx1 first-dose	557,037	85,392	<5	4.8	
BNT162b2 first-dose	1,720,473	93,33	5	5.8	0.87 (0.36 to 2.09)
BNT162b2 second-dose	1,144,392	68,071	<5	4.9	
mRNA-1723 second-dose	147,734	11,589	<5	0.6	
Transverse Myelitis					
BNT162b2 first-dose	1,720,543	93,334	<5	0.9	
**Results without requiring year of prior history**					
Bell's Palsy					
ChAdOx1 first-dose	594,588	89,329	27	82.7	0.33 (0.22 to 0.48)
ChAdOx1 second-dose	231,228	55,786	10	47.3	0.21 (0.11 to 0.39)
BNT162b2 first-dose	1,900,497	103,128	100	117.1	0.85 (0.70 to 1.04)
BNT162b2 second-dose	1,256,600	74,934	85	97.4	0.87 (0.71 to 1.08)
mRNA-1723 first-dose	245,672	17,731	14	15.3	0.92 (0.54 to 1.55)
mRNA-1723 second-dose	160,667	12,615	5	11.4	0.44 (0.18 to 1.06)
Ad26.COV2.S first-dose	122,497	5,319	6	5.3	1.14 (0.51 to 2.53)
SARS-CoV-2 positive test	289,417	66,921	93	54.9	1.69 (1.38 to 2.07)
Encephalomyelitis					
ChAdOx1 first-dose	595,068	89,396	5	6.1	0.82 (0.34 to 1.97)

BNT162b2 first-dose	1,902,480	103,244	9	11.7	0.77 (0.40 to 1.48)
BNT162b2 second-dose	1,258,126	75,03	4	10.3	0.39 (0.15 to 1.03)
mRNA-1723 first-dose	245,949	17,752	<5	1.2	
Ad26.COV2.S first-dose	122,615	5,324	<5	0.4	
SARS-CoV-2 positive test	289,761	67,006	17	4.5	3.75 (2.33 to 6.04)
Guillain Barré Syndrome					
ChAdOx1 first-dose	595,085	89,397	<5	5.0	
BNT162b2 first-dose	1,902,494	103,245	5	6.3	0.79 (0.33 to 1.90)
BNT162b2 second-dose	1,258,125	75,03	<5	5.4	
mRNA-1723 second-dose	160,885	12,633	<5	0.7	
SARS-CoV-2 positive test	289,78	67,011	17	2.9	5.93 (3.69 to 9.54)
Transverse Myelitis					
BNT162b2 first-dose	1,902,581	103,25	<5	1.0	
mRNA-1723 second-dose	160,894	12,634	<5	0.1	

Table 7. Sensitivity analyses for the self-controlled case series (SCCS) excluding individuals infected with SARS-CoV-2 prior to vaccination. Incidence rate ratios (95% confidence intervals) for COVID-19 vaccination or infection and the occurrence of Bell's Palsy.

	**CPRD AURUM**				**SIDIAP**					
	**ChAdOx1**		**BNT162b2**		**ChAdOx1**		**BNT162b2**		**mRNA-1273**	
	Number of events	IRR	Number of events	IRR	Number of events	IRR	Number of events	IRR	Number of events	IRR
Baseline	6935	Ref	4375	Ref	4455	Ref	8438	Ref	3695	Ref
−21 to −1 days pre-exposure	74	0.66 (0.52 to 0.83)	39	0.76 (0.54 to 1.03)	11	0.44 (0.23 to 0.75)	64	0.67 (0.52 to 0.85)	11	0.91 (0.47 to 1.56)
Day 0	5	0.91 (0.32 to 1.95)	<5		<5		<5		<5	
Days 1 - 7 post exposure	24	0.61 (0.40 to 0.89)	13	0.75 (0.41 to 1.23)	5	0.60 (0.21 to 1.29)	20	0.63 (0.39 to 0.95)	<5	
Day 8 - 14 post exposure	31	0.80 (0.55 to 1.12)	16	0.92 (0.54 to 1.45)	5	0.60 (0.22 to 1.30)	27	0.88 (0.59 to 1.26)	<5	
Days 15 - 21 post exposure	40	1.05 (0.75 to 1.41)	15	0.85 (0.48 to 1.36)	11	1.33 (0.69 to 2.29)	22	0.78 (0.50 to 1.16)	<5	
Days 1-21 post exposure	95	0.82 (0.66 to 1.00)	44	0.84 (0.61 to 1.12)	21	0.84 (0.53 to 1.26)	69	0.76 (0.60 to 0.96)	*	0.95 (0.49 to 1.63)

CPRD: Clinical Practice Research Datalink; SIDIAP: Information System for Research in Primary Care. Events with less than 5 occurrences have been omitted for privacy reasons.

Table 8. Cases of immune-mediated neurological events with hospitalisation in SIDIAP between 1 January 2017 to 30 June 2021

	Number of cases	Cases with hospitalisation (Day 0) (row %)	Cases with hospitalisation (Day 0 to Day 3) (row %)
Bell’s palsy	19,938	6,687 (33.5 %)	7,166 (35.9 %)
Encephalomyelitis	1,603	1,295 (80.8 %)	1,384 (86.3%)
Guillain Barre syndrome	1,018	704 (69.2 %)	801 (78.7%)
Transverse myelitis	193	106 (54.9 %)	114 (59.1%)

SIDIAP: Information System for Research in Primary Care

Table 9. Cases of death following immune-mediated neurological events between 1 January 2017 to 30 June 2021

	**Number of cases**	**Cases of death (Day 0 to Day 7) (row %)**
**CPRD AURUM**		
Bell's palsy	107,652	45 (0.4%)
Encephalomyelitis	9,253	32 (0.3%)
Guillain Barre syndrome	7,474	20 (0.3%)
Transverse myelitis	4,465	<5
**SIDIAP**		
Bell's palsy	19.938	53 (0.3%)
Encephalomyelitis	1,603	6 (0.4%)
Guillain Barre syndrome	1,018	<5
Transverse myelitis	193	0

CPRD: Clinical Practice Research Datalink; SIDIAP: Information System for Research in Primary Care. These counts were calculated by taking all persons who experienced any of the studied immune-mediated neurological disorders from 1 January 2017 to end of data availability in the two databases.

Figure 1A. Time between end of study or censor date and occurrence of Bell's palsy from 1 January 2017 to end of data availability in CPRD AURUM

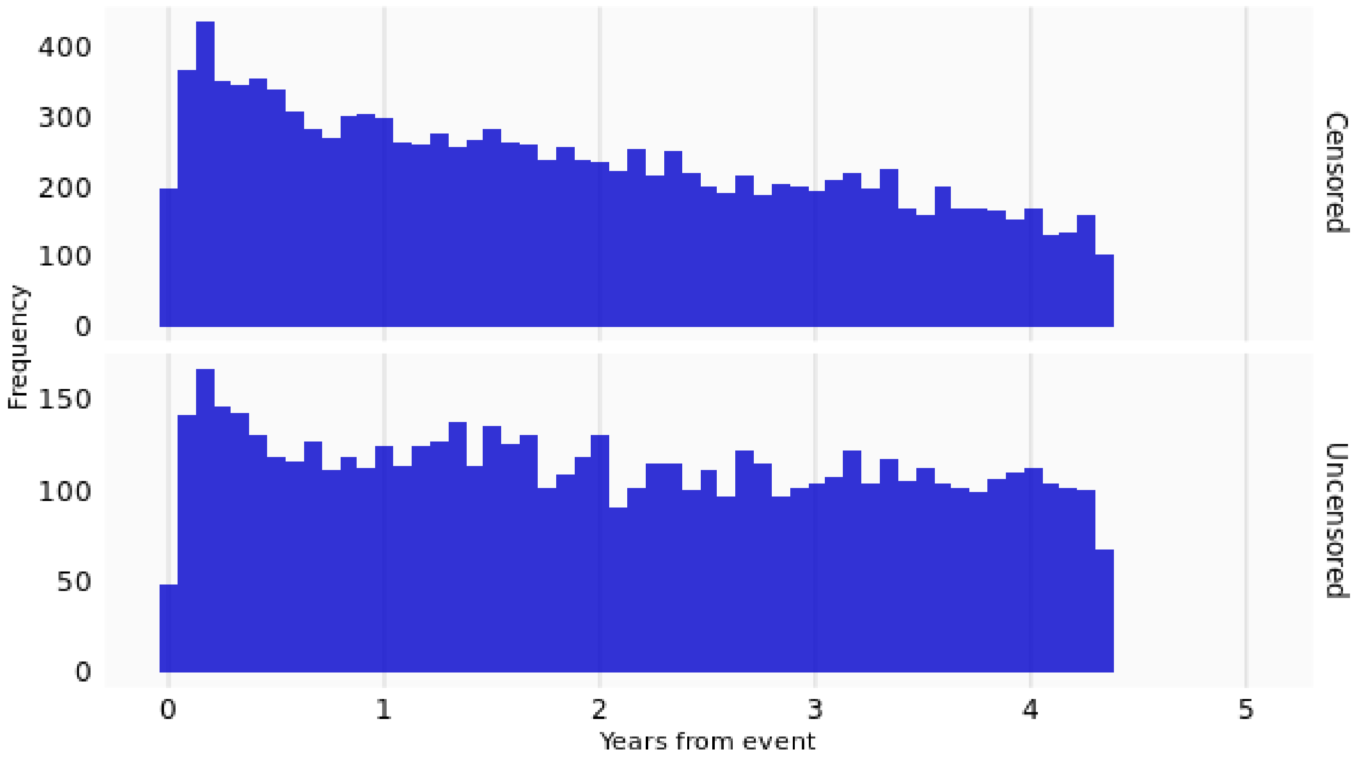

CPRD: Clinical Practice Research Datalink

Figure 1B. Time between end of study or censor date and occurrence of Bell's palsy from 1 January 2017 to end of data availability in SIDIAP

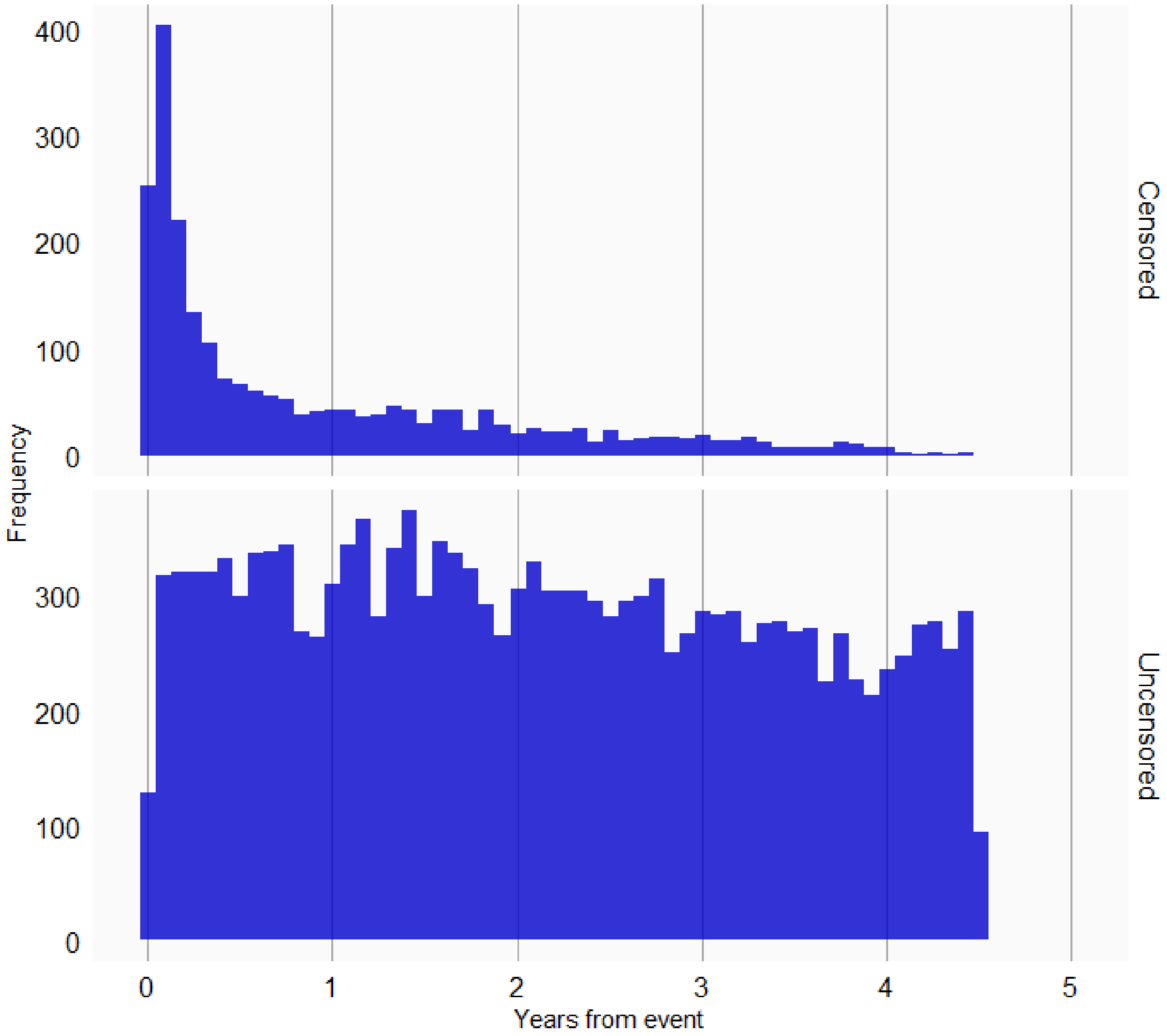

SIDIAP: Information System for Research in Primary Care

Figure 2. Incidence Rate Ratios (IRR) by age group and sex

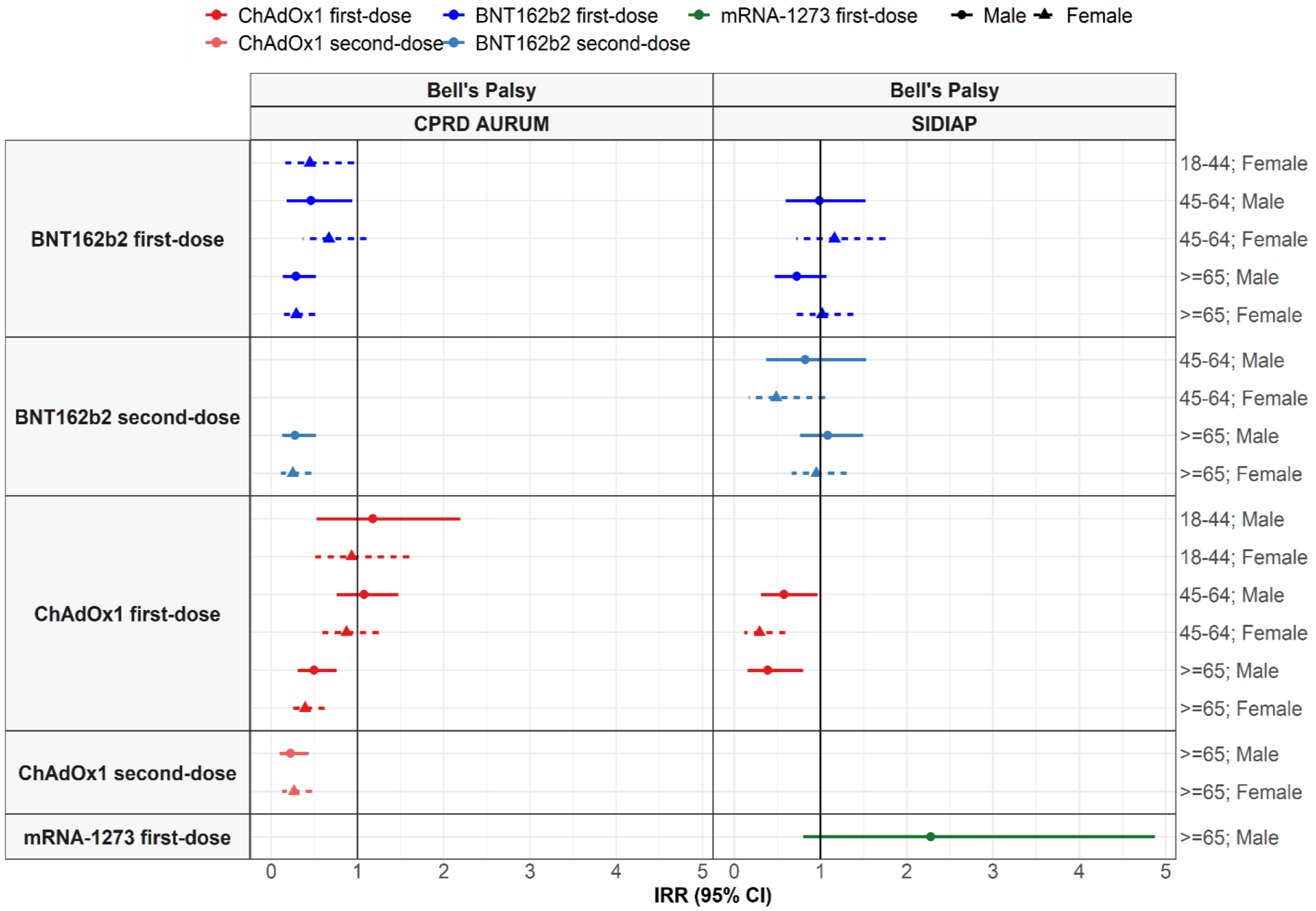

CPRD: Clinical Practice Research Datalink; SIDIAP: Information System for Research in Primary Care**.** Events with less than 5 occurrences have been omitted for privacy reasons. No events were observed for the omitted cohorts
